# Role of anti-*Pseudogymnoascus destructans* bacteria in cave ecosystems during bat hibernation in northeast China

**DOI:** 10.1128/aem.02214-25

**Published:** 2026-04-24

**Authors:** Haixia Leng, Xiaoyu Sun, Yingting Pu, Long Huang, Wentao Dai, Jiang Feng, Keping Sun

**Affiliations:** 1 Jilin Provincial Key Laboratory of Animal Resource Conservation and Utilization, Northeast Normal University 47821, Changchun, China; 2 Key Laboratory of Vegetation Ecology, Ministry of Education, Changchun, China; 3 Jilin Provincial International Cooperation Key Laboratory for Biological Control of Agricultural Pests, Jilin Agricultural University 85112, Changchun, China; Colorado School of Mines, Golden, Colorado, USA

**Keywords:** *Pseudogymnoascus destructans*, cave microbiota, volatile organic compounds, antifungal bacteria, bat hibernation

## Abstract

White-nose syndrome, caused by *Pseudogymnoascus destructans* (*Pd*), threatens multiple bat species. Bat skin plays a crucial role in defense against pathogens, especially during hibernation. Cave environments serve as both a source and reservoir of bat skin microbiota, being vital to defend against pathogen transmission and prevent pathogen invasion. In this study, we employed culture-independent and culture-dependent methods to analyze the environmental microbial community and anti-*Pd* bacteria on cave walls where bats hibernate. We found that cave wall environments sampled under different *Pd* detection statuses exhibited similar bacterial community structures. Both the different sampling times and areas influenced the beta diversity of the cave wall bacterial communities. The cave wall microbiota showed the ability to inhibit the *Pd* pathogen, as we isolated 19 genera of anti-*Pd* bacteria, 9 of which demonstrated novel inhibitory effects on *Pd* growth. Seven volatile organic compounds (VOCs) produced by these bacteria inhibited *Pd* growth at 100 mM concentration. Furthermore, amplicon sequence variants (ASVs) obtained through dependent culture accounted for 12.07–55.2% of the ASVs obtained through independent culture, with 11.21–34.4% of these ASVs exhibiting inhibitory effects on *Pd* growth. This study highlighted the resistance of cave wall microbiota to *Pd* and identified candidate strains for biological control of WNS in bat hibernacula.

**IMPORTANCE:**

Understanding the role of cave ecosystems in preventing disease invasion is essential, as caves where bats roost act as natural reservoirs of bacteria. This study focused on the cave walls where bats hibernate, examining the composition of bacterial communities during hibernation and identifying bacteria that inhibit the pathogen *Pseudogymnoascus destructans* (*Pd*), the pathogen responsible for white-nose syndrome (WNS). It was found that both the sampling times and areas influenced the microbial composition on the cave walls. The study identified 19 bacterial genera on the cave walls that effectively inhibited *Pd* growth, with some bacteria producing volatile organic compounds (VOCs) that further suppressed *Pd*. These findings provide valuable insights for environmental biocontrol strategies to combat WNS.

## INTRODUCTION

Caves, characterized by low light, limited nutrients, high humidity, and stable temperatures, provide unique habitats for diverse wildlife (1). Cave-dwelling bats, in particular, spend extended periods in these environments, especially during the winter months, when temperate bats hibernate for several months. In recent years, white-nose syndrome (WNS), caused by the fungal pathogen *Pseudogymnoascus destructans* (*Pd*), has led to significant declines in multiple bat species across North America (2, 3). While *Pd* has also been detected in bats in Asia, no bat mortality associated with the syndrome has been observed (4, 5). *Pd* thrives in the cold and humid conditions of caves, where it can grow, spread, and persist (6, 7).

Bats in hibernation are particularly susceptible to WNS (3, 8), with symptoms of infection including weight loss, dehydration, and electrolyte imbalance, which can ultimately lead to mortality (2). The primary mode of transmission for *Pd* is through direct contact between bats (9). Additionally, bats may also become infected with cave-sourced *Pd* during autumn and winter by coming into contact with contaminated surfaces (10). The prevalence of *Pd* within a cave influences the infection load and prevalence among bat populations during hibernation (11). However, different cave environments across regions exhibit distinct patterns of *Pd* transmission (11). In North America, *Pd* prevalence steadily increases each winter following its initial invasion and remains stable or slightly increases over the following summers (11). In contrast, cave environments in East Asia show lower *Pd* prevalence, with peaks occurring after bat hibernation ends, followed by clearance of the pathogen by the following summer, returning to minimal levels (11). The reasons for the lower *Pd* prevalence in East Asia remain unclear.

Cave-dwelling microorganisms play a crucial role in inhibiting the growth of *Pd* spores *in vitro* (12, 13). For instance, certain probiotic bacteria isolated from caves have been shown to efficiently inhibit *Pd* growth, such as *Acinetobacter*, *Pseudomonas*, and *Stenotrophomonas* (14). Some of these volatile organic compounds (VOCs) produced by certain antagonistic strains have been identified as inhibitors of *Pd* growth and may be considered for introduction into bat hibernation environments (14–19). Therefore, understanding the composition, dynamics, and potential of microbial communities on winter cave walls to inhibit *Pd* is crucial for guiding the development of field-based biological control methods. These strategies aimed to reduce the environmental reservoir of *Pd* in caves, which could contribute to the prevention and management of WNS.

This study focused on caves with low *Pd* load and prevalence in bat hibernacula during the winter. An integrated approach, combining both dependent and independent culture methods, was employed to explore the dynamics of bacterial communities on cave walls and their potential to inhibit *Pd*. The main objectives of the study were as follows: (i) to characterize changes in the microbial community on winter cave walls, particularly identifying antifungal genera and their relative abundance; (ii) to investigate the relationship between the composition of winter cave wall microorganisms and factors such as *Pd* detection status, sampling time, and sampling area; and (iii) to isolate bacterial strains from winter cave walls that exhibit resistance to *Pd* and evaluate the inhibitory effects of VOCs produced by these bacteria on *Pd*. Our findings identify bacterial strains and VOCs with potent *in vitro* anti-*Pd* activity, providing promising candidates for future evaluation as biological control tools against WNS.

## RESULTS

### Distribution and detection of *Pd* on cave walls

Of the 51 samples, 7 tested *Pd*-positive (Table S1), with an average *Pd* load of 1.22e^−5^ ± 1.32e^−5^ (mean ± standard deviation) ng and average Ct value of 38.96 ± 1.13. The *Pd* load in this study is markedly lower than reported from North America (1.46e^−5^–1.77e^−5^ ng) (Table S2) (9, 11). *Pd* prevalence peaked in December (29.4%), followed by February and March samples (Feb: 5.88%, Mar: 5.88%). Dark zone samples exhibited a higher fungal prevalence than twilight zone samples (dark zone: 15.38%, twilight zone: 8.3%). Given that only a single *Pd*-positive sample was found in the twilight zone, we focused the *Pd*-status comparison solely on the dark-zone subset to enable a clear interpretation of the differences.

### Winter cave wall bacterial composition and diversity using culture-independent methods

**Fig 1:**
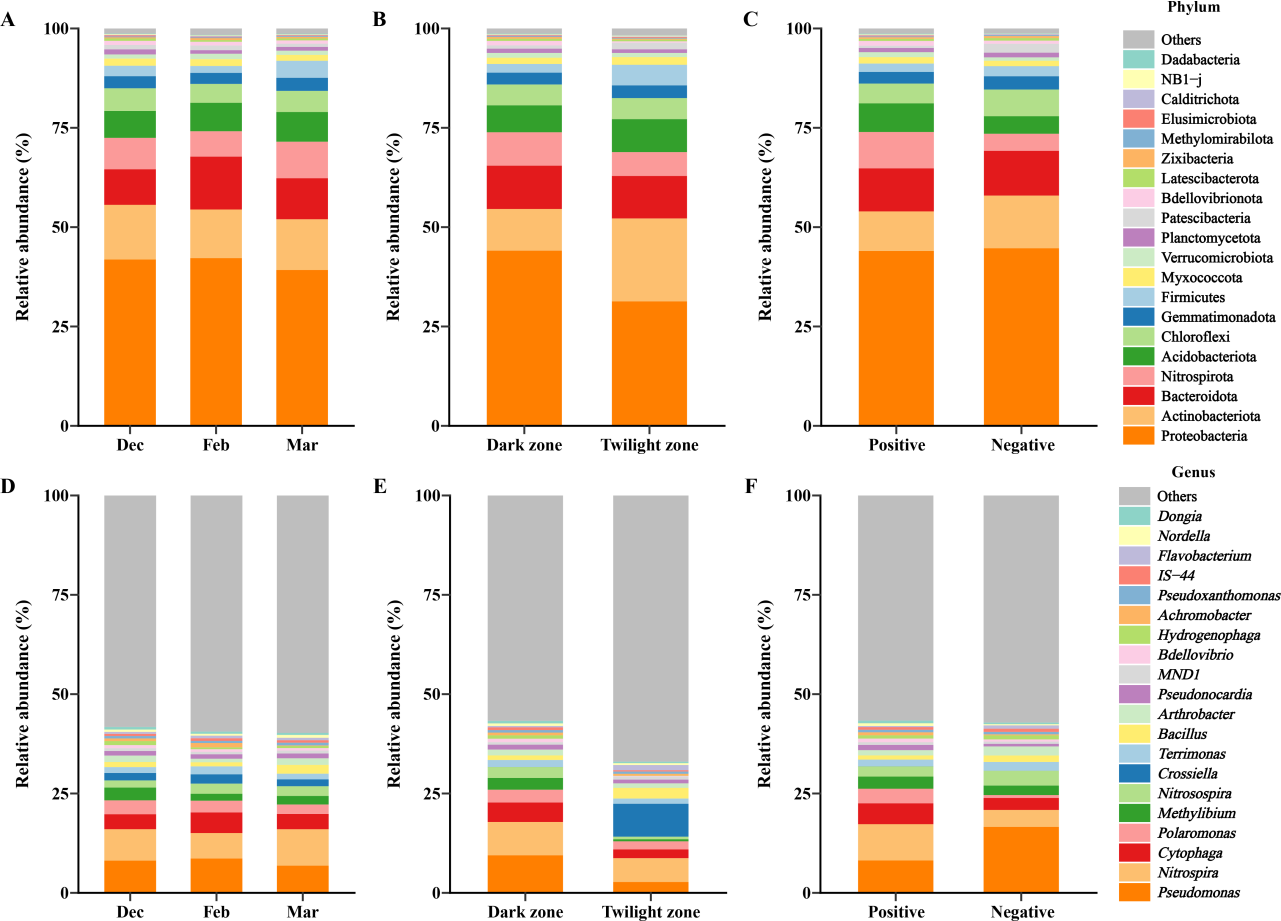
Species composition of the microbial communities in the cave walls. Average relative abundance at the phylum level across different sampling times (**A**), across different sampling areas (**B**), and based on *Pd* detection statuses (**C**). Average relative abundance of the top 20 bacterial genera across different sampling times (**D**), across different sampling areas (**E**), and based on *Pd* detection statuses (**F**).

The microbial composition of the cave wall comprised 20 phyla (Fig. 1A through C), with Proteobacteria, Actinobacteriota, Bacteroidota, Firmicutes, and Chloroflexi being the most predominant, accounting for more than 75% of the relative abundance. Across sampling time, area, and *Pd* detection status, Proteobacteria exhibited the highest average relative abundance (Fig. 1A through C). The MaAsLin3 analysis revealed that Fibrobacterota was significantly enriched in the twilight zone compared to the dark zone after adjusting for sampling time and *Pd* detection status (coef = 2.48, *q* = 0.037).

At the genus level, the microbial composition of the cave wall included several genera, with the relative abundance of the top 20 genera accounting for less than 50% of the total (Fig. 1D through F). Differential abundance analysis using MaAsLin3 revealed significant shifts in microbial communities associated with the sampling area and *Pd* detection status. The twilight zone exhibited a distinct microbial signature, with four genera significantly enriched (*Smaragdicoccus*: coef = 2.84, *q* < 0.001; *Rhodococcus*: coef = 1.97, *q* = 0.036; *Bauldia*: coef = 1.25, *q* = 0.073; *Luteolibacter*: coef = 2.44, *q* = 0.073) and four genera being significantly depleted (*Methylibium*: coef = −3.51, *q* < 0.001; *Hydrogenophaga*: coef = −3.43, *q* < 0.001; *Sandaracinus*: coef = −1.54, *q* = 0.026; AKYG587: coef = −2.19, *q* = 0.036). Additionally, the nitrifying genus *Nitrospira* (coef = −1.53, *q* = 0.046) was significantly depleted in positive samples.

**Fig 2:**
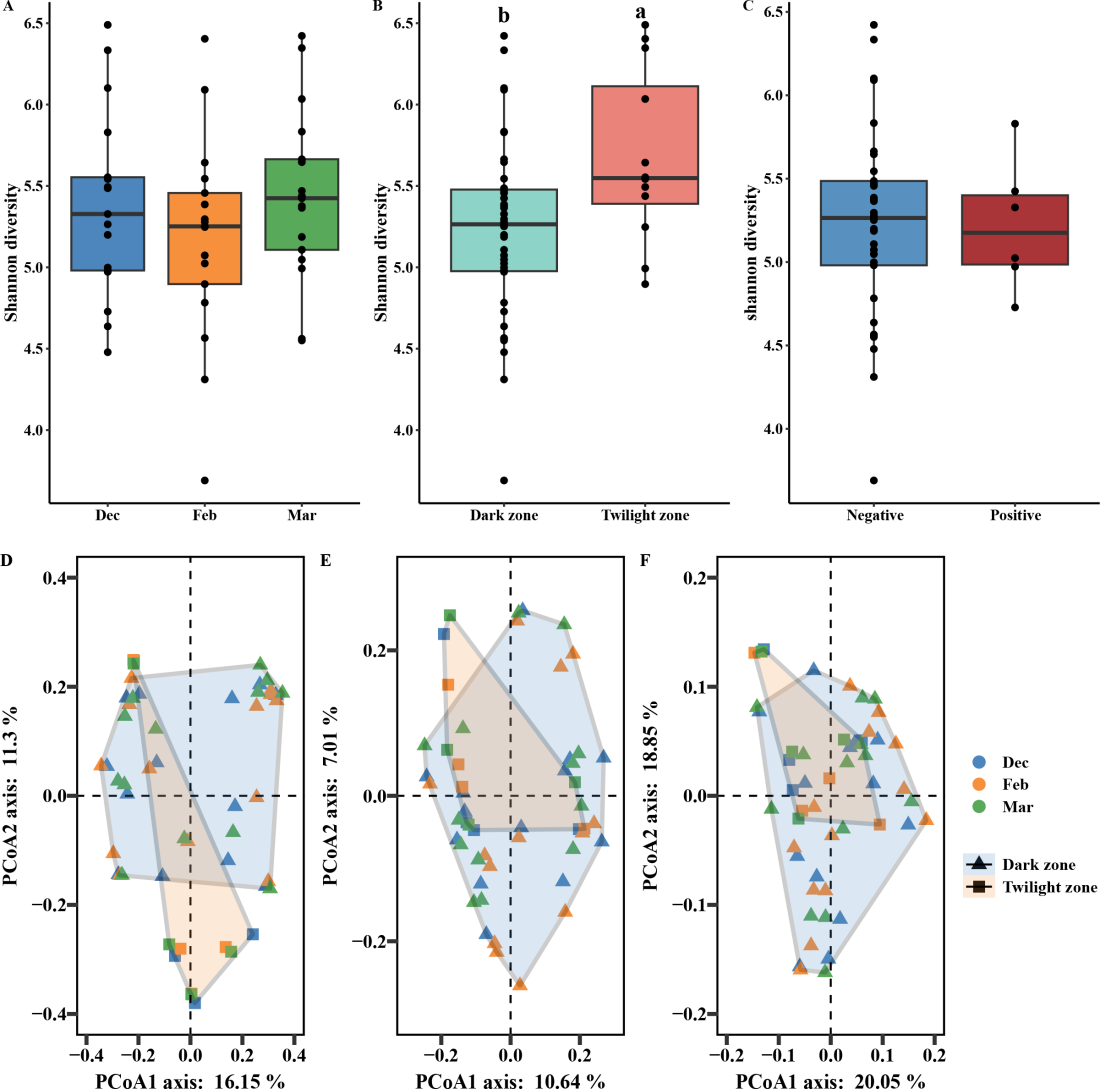
Alpha and beta diversity of the cave wall. Shannon diversity across different sampling times (**A**), sampling areas (**B**), and *Pd* detection statuses (**C**), with letters indicating significant differences between groups. PCoA analyses based on Bray–Curtis dissimilarity (**D**), weighted UniFrac distance (**E**), and unweighted UniFrac distance (**F**) of the cave wall microbial community.

To determine whether temporal variation, spatial heterogeneity, or *Pd* presence influenced overall community diversity, we compared alpha diversity metrics across sampling times, sampling areas, and *Pd* detection statuses. Across sampling times, the Chao1 richness, Shannon diversity, and Faith’s phylogenetic diversity indices showed no significant differences (Shannon diversity: ANOVA, F = 0.98, *P* = 0.38; Chao1 richness: Kruskal-Wallis test, χ^2^ = 3.57, *P* = 0.168; Faith’s phylogenetic diversity: χ^2^ = 4.806, *P* = 0.09) (Fig. 2A; Fig. S1A and D). In contrast, alpha diversity between different sampling areas for Shannon diversity and Chao1 richness (Shannon diversity: *t*-test, *t* = −2.46, *P* = 0.02; Chao1 richness: Mann-Whitney *U* test, *W* = 132, *P* =0.024) exhibited significant differences, but not for Faith’s phylogenetic diversity (Mann-Whitney *U* test, *W* = 171, *P* = 0.65) (Fig. 2B; Fig. S1B and E). No significant differences in alpha diversity were observed between different *Pd* detection statuses (Shannon diversity: *t*-test, t = 0.03, *P* = 0.976; Chao1 richness: Mann-Whitney *U* test, *W* = 119, *P* = 0.345; Faith’s phylogenetic diversity: Mann-Whitney *U* test, *W* = 88, *P* = 0.077) (Fig. 2C; Fig. S1C and F).

To evaluate whether differences in beta diversity were influenced by unequal within-group dispersion, we assessed community dispersion using permutational analysis of multivariate dispersions (PERMDISP). PERMDISP analysis revealed homogeneous community dispersion across both sampling time (Bray–Curtis dissimilarity: pseudo-F_2,48_ = 0.09, *P* = 0.917; unweighted UniFrac distance: pseudo-F_2,48_ = 2.401, *P* = 0.101; weighted UniFrac distance: pseudo-F_2,48_ = 0.332, *P* = 0.719), sampling area (Bray–Curtis dissimilarity: pseudo-F_1,49_ = 1.093, *P* = 0.301; unweighted UniFrac distance: pseudo-F_1,49_ = 1.911, *P* = 0.173; weighted UniFrac distance: pseudo-F_1,49_ = 0.326, *P* = 0.571), and *Pd* detection status (Bray–Curtis dissimilarity: pseudo-F_1.37_ = 2.336, *P* = 0.135; unweighted UniFrac distance: pseudo-F_1.37_ = 1.055, *P* = 0.311; weighted UniFrac distance: pseudo-F_1.37_ = 1.055, *P* = 0.311), indicating that permutational multivariate analysis of variance (PERMANOVA) results were not driven by heterogeneity of dispersion (Fig. S2).

Beta diversity measures among different sampling times (Bray–Curtis dissimilarity: pseudo-F_2,46_ = 0.589, *P* < 0.001; weighted UniFrac distance: pseudo-F_2,46_ = 0.786, *P* < 0.001; unweighted UniFrac distance: pseudo-F_2,46_ = 0.021, *P* < 0.001). Sampling areas significantly affected microbial community composition based on Bray–Curtis dissimilarity (pseudo-F_1,46_ = 3.946, *P* = 0.013) and unweighted UniFrac distance (pseudo-F_1,46_ = 2.267, *P* = 0.036), but not on weighted UniFrac distance (pseudo-F_1,46_ = 4.325, *P* = 0.056). With the unweighted UniFrac distance explaining the highest variance on the PCoA1 and PCoA2 axes (38.9%) (Fig. 2D through F). In contrast, no significant differences in beta diversity measures were found between negative and positive samples (Bray–Curtis dissimilarity: pseudo-F_1,46_ = 1.949, *P* = 0.307; weighted UniFrac distance: pseudo-F_1,46_ = 2.118, *P* = 0.510; unweighted UniFrac distance: pseudo-F_1,46_ = 1.622, *P* = 0.450) (Table S3).

### Potentially anti-*Pd* bacteria in the winter cave walls using culture-independent methods

**Fig 3:**
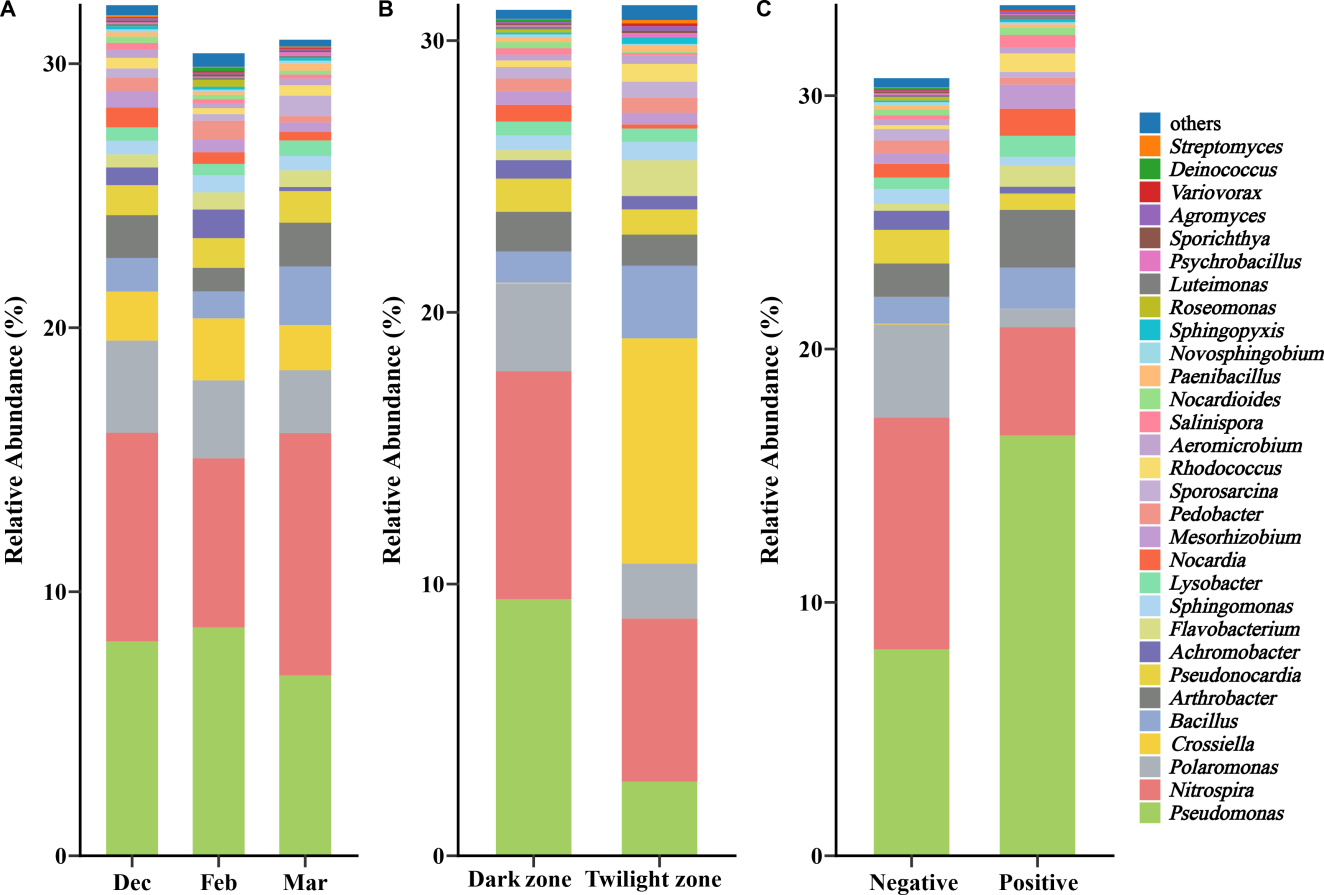
Potential anti-*Pd* genus level composition of cave wall microorganisms. Composition across different sampling times (**A**), across different sampling areas (**B**), and based on different *Pd* detection statuses (**C**).

Based on the sequence from published literature on potential fungal pathogen inhibition, a total of 1,315 amplicon sequence variants (ASVs), belonging to 50 families and 72 genera, were identified by matching ASVs with relative abundance > 0.01% from culture-independent sequencing data. Similarity analysis of the 31 matched genera, conducted through file retrieval, revealed that the sequence similarity ranged from 95.02% to 100% (Table S4). Among the top 30 genera based on relative abundance, 1,123 ASVs were matched, belonging to 21 families (Fig. 3). Several of these ASVs showed 100% similarity across 9 families and 13 genera (Table S5). In different time groupings, *Pseudomonas*, *Nitrospira*, *Polaromonas*, and *Crossiella* exhibited higher relative abundances, while *Achromobacter* (Krustal-Willis: χ^2^ = 14.06, *P* < 0.001), *Deinococcus* (Krustal-Willis: χ^2^ = 6.24, *P* = 0.044), *Paenibacillus* (Krustal-Willis: χ^2^ = 9.998, *P* =0.007), *Pedobacter* (Krustal-Willis: χ^2^ = 11.65, *P* = 0.003), and *Psychrobacillus* (Krustal-Willis: χ^2^ = 6.356, *P* = 0.042) showed significant differences. The dark zone group had a significantly higher average relative abundance of *Agromyces* (Mann-Whitney *U*: *W* = 122, *P* = 0.013), *Lysobacter* (Mann-Whitney *U*: *W* = 337, *P* = 0.021), *Nocardioides* (Mann-Whitney *U*: *W* = 377, *P* = 0.001), *Pseudomonas* (Mann-Whitney *U*: *W* = 346, *P* = 0.010), and *Rhodococcus* (Mann-Whitney *U*: *W* = 69, *P* < 0.001) compared to the twilight zone group. Additionally, the dark zone group exhibited a significantly lower average relative abundance of *Novosphingobium* (Mann-Whitney *U*: *W* = 350, *P* = 0.01) and *Streptomyces* (Mann-Whitney *U*: *W* = 54, *P* < 0.001) compared to the twilight zone group (Table S6). Additionally, the negative group had a significantly higher average relative abundance of *Achromobacter* (Mann-Whitney *U*: *W* = 151, *P* = 0.043), *Nitrospira* (Mann-Whitney *U*: *W* = 162, *P* = 0.012), *Polaromonas* (Mann-Whitney *U*: *W* = 169, *P* = 0.005), and *Rhodococcus* (Mann-Whitney *U*: *W* = 34, *P* = 0.012) compared to the positive group. Furthermore, the negative group had a significantly lower average relative abundance of *Luteimonas* (Mann-Whitney *U*: *W* = 46, *P* = 0.032) compared to the positive group (Table S6).

### Isolation and identification of anti-*Pd* bacteria in winter cave walls using culture-dependent methods

**Fig 4:**
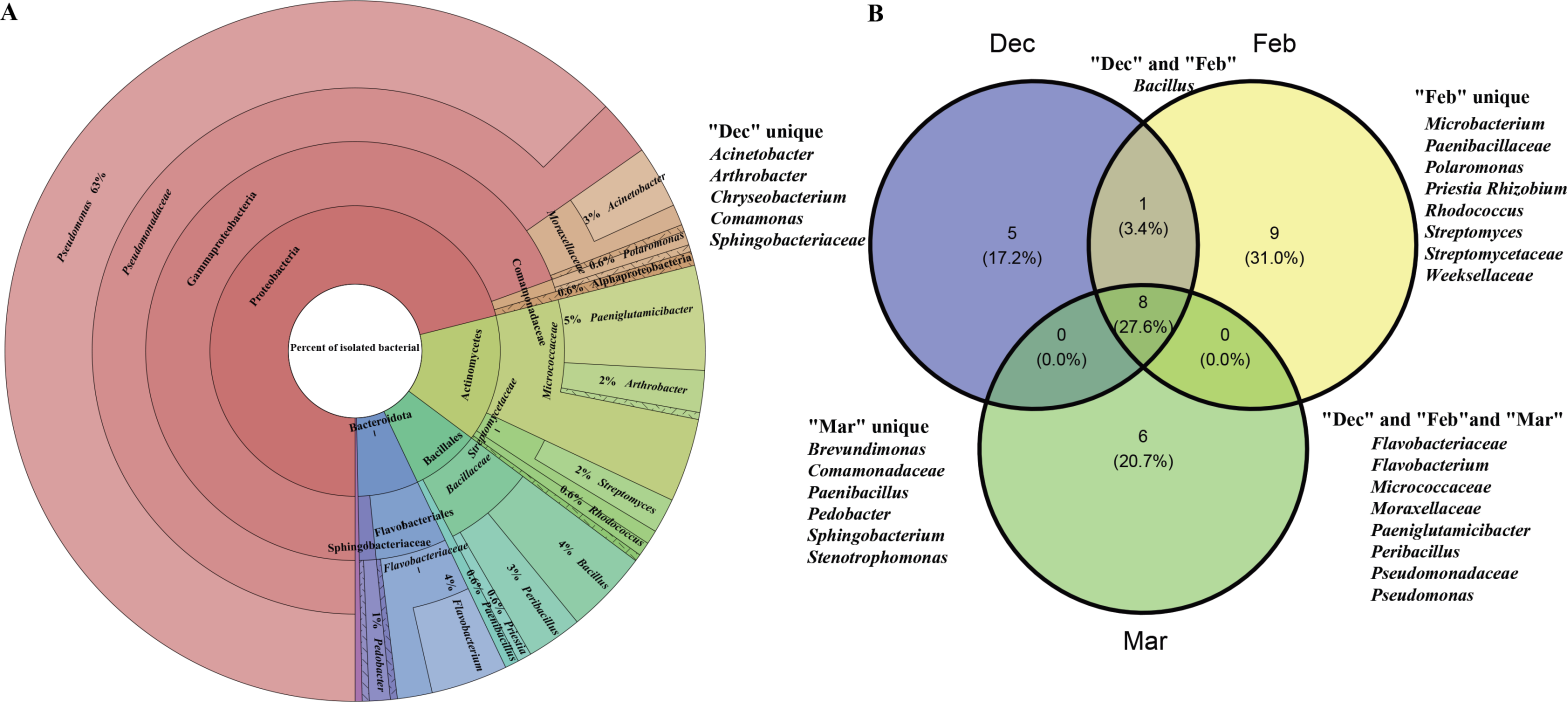
Composition of bacteria isolated and cultured from cave wall swabs. (**A**) Species composition, where the circles from inner to outer represent the levels of phylum, class, order, family, and genus. (**B**) Venn diagram of species composition in three different times.

A total of 311 bacterial isolates were obtained from 51 cave wall samples collected over three winter periods, representing 4 phyla and 21 genera (Fig. 4; Table S7). The predominant phyla identified through culture-dependent isolation were Proteobacteria, Actinobacteriota, and Bacteroidota, which were consistent with the results from culture-independent data. Notably, *Pseudomonas* was the most abundant genus, accounting for 63% of the isolates at the genus level (Fig. 4A). The genera shared across the three time periods contributed to 27.6% of the total isolates. These genera included *Flavobacterium*, *Paeniglutamicibacter*, *Peribacillus*, and *Pseudomonas*, as well as members from four families: *Flavobacteriaceae*, *Micrococcaceae*, *Moraxellaceae*, and *Pseudomonadaceae* (Fig. 4B).

**Fig 5:**
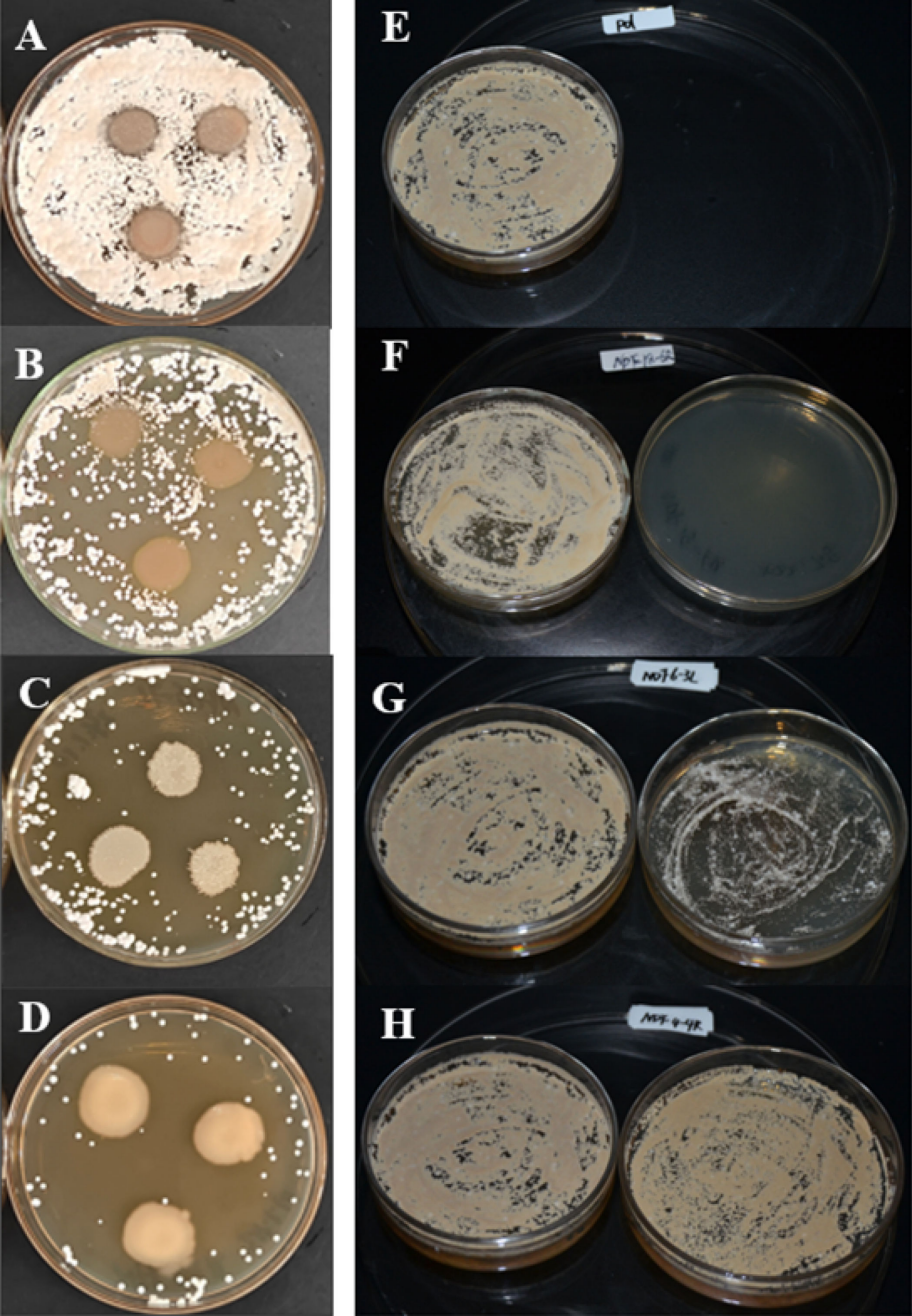
Inhibitory effect of two types of inhibition experiments on *Pd* growth. (**A–D**) Contact experiments: (**A**) no inhibition, (**B**) weak inhibition, (**C**) moderate inhibition, and (**D**) strong inhibition. (**E–H**) Non-contact experiments: (**E**) *Pd* control, (**F**) complete or near-complete inhibition (>85% inhibition), (**G**) significant inhibition (50–85%), and (**H**) negligible or no inhibition (≤50% inhibition).

In the contact inhibition experiment, only 4.18% of the strains showed complete inhibitory effects, while the largest proportion (65.59%) showed no inhibitory effects (Tables S8 and S9; Fig. 5A through D). A total of 13 strains that strongly inhibited the growth of *Pd* were identified, belonging to three genera (*Bacillus*, *Pseudomonas*, and *Pedobacter*) and one family (*Micrococcaceae*). Of these, one strain was identified at the species level: *Ba. mycoides*. In addition, 94 strains displayed moderate to weak inhibition, accounting for 30.22% of the total isolates (Table S8).

In the non-contact inhibition experiment, a total of 142 strains were identified that either completely or nearly completely inhibited the growth of *Pd*, accounting for 45.66% of all isolated strains (Tables S8 and S9; Fig. 5F). These strains were classified into 19 genera: *Acinetobacter*, *Arthrobacter*, *Bacillus*, *Brevundimonas*, *Chryseobacterium*, *Comamonas*, *Flavobacterium*, *Paenibacillus*, *Paeniglutamicibacter*, *Pedobacter*, *Peribacillus*, *Polaromonas*, *Pseudarthrobacter*, *Pseudomonas*, *Rhizobium*, *Rhodococcus*, *Sphingobacterium*, *Stenotrophomonas*, and *Streptomyces*. Additionally, 11 strains were classified at the family level: *Flavobacteriaceae*, *Micrococcaceae*, *Pseudomonadaceae*, and *Weeksellaceae*. Three strains were identified at the species level as *Ps. vancouverensis* and *Ba. mycoides*. Moreover, 32 strains exhibited significant inhibitory effects on *Pd*, representing 10.29% of all isolated strains (Tables S8 and S9; Fig. 5G). One strain was identified at the species level as *Priestia aryabhattai B8W22*.

In both contact and non-contact inhibition experiments, 16 taxa were found to exhibit inhibitory effects on *Pd*, including 13 genera (*Arthrobacter*, *Pseudomonas*, *Comamonas*, *Paenibacillus*, *Flavobacterium*, *Peribacillus*, *Brevundimonas*, *Bacillus*, *Paeniglutamicibacter*, *Pedobacter*, *Polaromonas*, *Stenotrophomonas*, and *Streptomyces*) and 3 families (*Micrococcaceae*, *Flavobacteriaceae*, and *Pseudomonadaceae*) accounting for 61.54% of the total. The non-contact inhibition experiment uniquely identified seven additional inhibitory genera (*Acinetobacter*, *Chryseobacterium*, *Priestia*, *Pseudarthrobacter*, *Rhizobium*, *Rhodococcus*, and *Sphingobacterium*) and two families (*Streptomycetaceae*, *Weeksellaceae*). The contact inhibition experiment uniquely featured family *Moraxellaceae*, accounting for 3.85% of the total (Fig. S3). Three strains from the family *Micrococcaceae*, *Pseudomonas*, and strains of *Ba. mycoides* exhibited complete inhibition of *Pd* in both inhibition experiments. The proportion of strains that completely or nearly completely inhibited *Pd* was highest in the non-contact inhibition experiment (Table S8).

Among the ASVs with a relative abundance greater than 0.01% in culture-independent method, 626 ASVs corresponding to 19 genera from the culture-dependent method were identified. These matched ASVs accounted for 5.29% of the ASVs with a relative abundance > 0.01% in the culture-independent method. Notably, *Chryseobacterium*, *Comamonas*, *Peribacillus*, *Priestia*, *Pseudarthrobacter*, and *Rhizobium* were absent in the culture-dependent data. From the 311 ASVs identified in the culture-dependent data, 38, 69, and 21 matched ASVs were found in December, February, and March, respectively, with sequence alignment similarities of 100% (Table S10). The majority of these matched ASVs in culture-dependent ASVs were found to have inhibitory effects on *Pd*. Although the proportion of culture-dependent ASVs in the culture-independent ASVs was not high, their relative abundance ranged from 12.07% to 55.2%. In February, the relative abundance of ASVs with inhibitory effects was the highest, at 34.4% (Fig. S4).

### Identification of VOCs using culture-dependent methods

**Fig 6:**
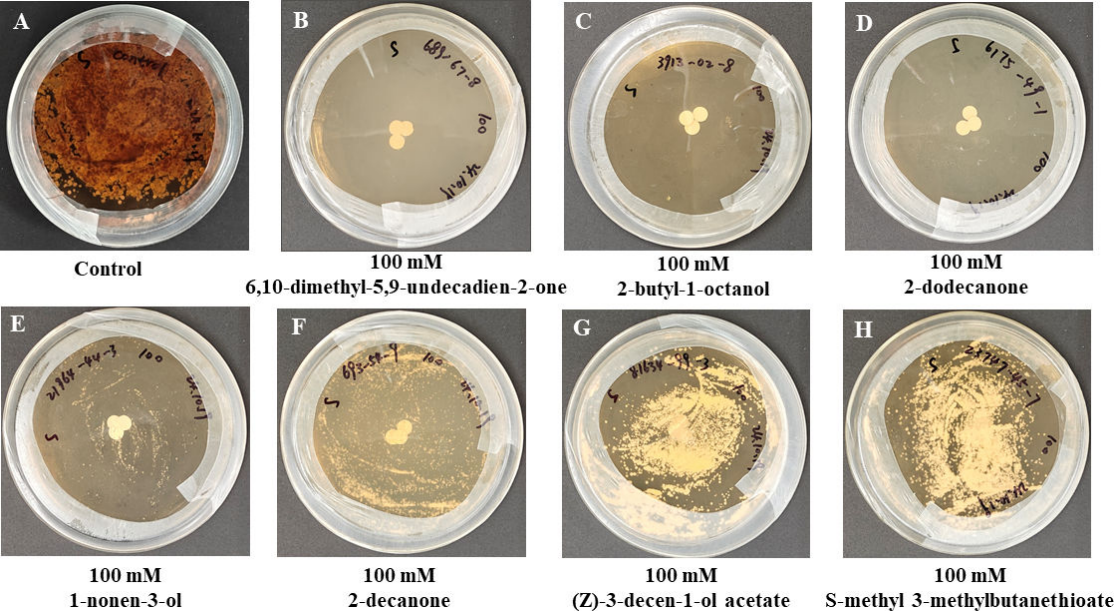
Seven VOCs effectively inhibit *Pd*. The blank control was co-cultured with *Pd* and 95% ethanol for 14 days (13°C, 90% relative humidity). (**A**) Control, (**B**) 6,10-dimethyl-5,9-undecadien-2-one, (**C**) 2-butyl-1-octanol, (**D**) 2-dodecanone, (**E**) 1-nonen-3-ol, (**F**) 2-decanone, (**G**) (Z)-3-decen-1-ol acetate, and (**H**) S-methyl 3-methylbutanethioate.

**Fig 7:**
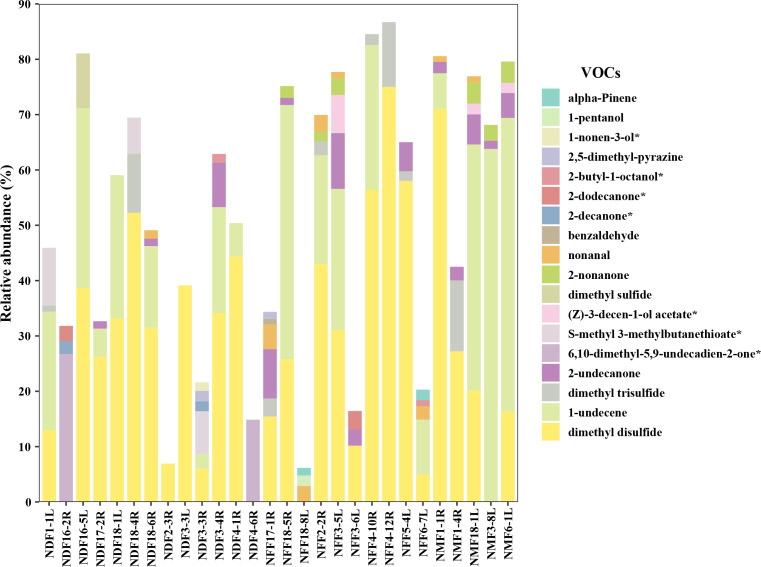
Relative abundance of VOCs with inhibitory activity against *Pd*. Compounds marked with an asterisk (*) were identified as inhibitory in this study, while the remaining compounds have been reported in the literature (Table S13).

To demonstrate complete inhibitory effects in spatial challenge experiments, VOCs from 13 bacterial genera were detected: *Arthrobacter*, *Acinetobacter*, *Bacillus*, *Chryseobacterium*, *Comamonas*, *Flavobacterium*, *Paeniglutamicibacter*, *Peribacillus*, *Pseudarthrobacter*, *Pseudomonas*, *Rhodococcus*, *Sphingobacterium*, and *Streptomyces*. Butylated hydroxytoluene, heptadecane, 6,10,14-trimethyl-2-pentadecanone, dimethyl disulfide, 1-undecene, (E)-2-decen-1-ol, nonanal, 2-undecanone, and chlorobenzene were shared by at least five genera (Fig. S5; Table S11). By analyzing VOCs with relative abundances exceeding 1%, we found that the primary VOCs produced by different genera that completely inhibit *Pd* growth are distinct. We selected 16 VOCs with relatively high abundances for further *Pd* inhibition experiments (Table S12). The results showed that 100 mM of 6,10-dimethyl-5,9-undecadien-2-one, 2-butyl-1-octanol, and 2-dodecanone completely inhibit *Pd* growth (Fig. 6B through D). Meanwhile, 1-nonen-3-ol, 2-decanone, (Z)−3-decen-1-ol acetate, and S-methyl 3-methylbutanethioate exhibited significant inhibitory effects on *Pd* (Fig. 6E through H). In addition to these VOCs, 11 others have also been reported in the literature to inhibit *Pd* (Table S13), with relative abundances ranging from 6.61% to 86.71% (Fig. 7; Table S11). This suggests that even VOCs present at relatively low abundances can inhibit *Pd*.

## DISCUSSION

In this study, cave environmental *Pd* loads remained consistently low and were lower than those in North America, aligning with the more severe and widespread impacts of WNS observed there (20). However, infection prevalence peaked in December. This seasonal pattern is consistent with some reports from North America (9) but differs from established long-term trends (21). Such discrepancies may arise from variations in environmental conditions, host populations, or sampling methodologies. Here, we examine the potential reasons for the low *Pd* loads in cave environments, as related to cave microorganisms.

Both culture-independent and culture-dependent methods confirmed that the bacteria from cave surfaces predominantly belonged to the phyla Proteobacteria, Bacteroidota, and Actinobacteriota, indicating the widespread and abundant presence of these phyla on cave walls, which play crucial roles in cave ecosystems, such as degrading organic compounds and enhancing nutrient availability (22). Throughout winter, alpha diversity on cave walls remained stable, likely due to the generally stable cave environments (23). However, significant variations in microbial composition and structure exist between the twilight zone (42.22–139.85 m from the cave entrance), which experiences greater temperature fluctuations, and the dark zone (146.21–223.97 m), which remains more stable. The twilight zone may support more taxa from external environments, a pattern observed in other caves (24, 25). This phenomenon may be attributed to external factors, such as water, wind, soil, and animal activity, influencing the twilight zone (24), while deeper zones are more dispersal-limited and exhibit distinct community composition and metabolic traits (25).

*Pseudomonas* was identified as a dominant genus in cave environments through both culture-independent and culture-dependent methods, with many strains showing inhibitory effects on *Pd. Pseudomonas* has been consistently detected in winter roosting substrates, bat fur, and guano, all exhibiting anti-*Pd* properties *in vitro* (26–29). Compounds produced by *Pseudomonas*, such as phenazines (18), phenylpyrrole derivatives (30), pyrrolnitrin (31), and hydrolytic enzymes (32), have broad antibacterial effects that inhibit fungal pathogens. In this study, a higher relative abundance of *Pseudomonas* was found in positive samples compared to negative ones, supporting previous bat skin microbiota findings (33). This suggests that *Pseudomonas* thrives in caves alongside *Pd*. Some *Pseudomonas* strains inhibit *Pd* growth *in vitro* (18, 28, 34, 35). Additionally, *Pseudomonas* was more abundant in the cave’s dark zone than in the twilight zone, likely due to direct contact between bat skin microbes and cave walls. Cave environments may serve as a source of anti-*Pd* bacteria that can colonize bat skin (34), linking microbial communities on bat skin to those in nearby substrates. Bacteria from bats may exchange organisms with cave wall microbes through direct contact (33). Although *Pseudomonas* can be a dermatological pathogen (36, 37), no harmful effects on bat skin have been observed. In addition to *Pseudomonas*, other antifungal genera, such as *Acinetobacter*, *Arthrobacter*, *Bacillus*, *Rhodococcus*, and *Serratia*, have been found in bat-inhabited cave environments (38). Among these, *Ba. humi* and *Ba. eiseniae* isolated from caves have shown effectiveness against pathogens, such as *Salmonella typhi* and *Staphylococcus aureus* (39). Additionally, species such as *Ba. subtilis*, *Ps. moraviensis*, *Arthrobacter humicola*, and *St. laeteviolaceus*, isolated from various environments (e.g., soil, wood, air), have completely inhibited *Pd* growth *in vitro* (14).

In this study, 19 genera of anti-*Pd* bacteria were isolated from the cave walls, indicating the cave environment’s potential to inhibit pathogens. Among these, *Pseudomonas*, *Rhodococcus*, *Arthrobacter*, *Bacillus*, *Sphingobacterium*, *Streptomyces*, and *Rhizobium* have been confirmed to inhibit *Pd* growth (17, 18, 28, 34). In addition, new isolates from the cave walls, including *Acinetobacter*, *Brevundimonas*, *Flavobacterium*, *Microbacterium*, *Pedobacter*, *Peribacillus*, *Paeniglutamicibacter*, *Pseudarthrobacter*, and *Sphingobacterium*, also demonstrated anti-*Pd*. Notably, *Acinetobacter*, *Brevundimonas*, *Flavobacterium*, *Microbacterium*, *Pedobacter*, *Paeniglutamicibacter*, and *Rhizobium* possess additional antifungal effects (38). *Acinetobacter* isolated from amphibian skin not only inhibits *Bd* growth but also reduces *Botrytis cinerea* activity by regulating plant responses (40). *Brevundimonas* found in *Aconitum vilmorinianum* Kom. (used for treating rheumatism) is believed to enhance the effects of aconitine, which has analgesic and anti-inflammatory properties (41). *Flavobacterium*, commonly found in soil and marine environments, can inhibit fungal spore formation and reproduction, showing antifungal activity (42). *Pedobacter*, isolated from amphibian skin, exhibits antifungal properties *in vitro* (43–45), suggesting it could control fungal pathogens, such as *Pd*, making it a candidate of interest for bat health. While *Sphingobacterium* from amphibian skin did not show resistance to *Bd* (46–49), our study detected *Paeniglutamicibacter* in the cold environments of bat hibernacula. Although some *Paeniglutamicibacter* species can degrade phenanthrene-containing pollutants (50), their ability to inhibit pathogens has not been reported.

Microbial VOCs, characterized by low molecular weight, high volatility, and lipophilicity, are crucial agents of microbial antagonism in the biological control (51). In this study, non-contact inhibition assays revealed more bacterial strains inhibiting *Pd* than contact inhibition assays, emphasizing the significance of microorganism-produced VOCs in suppressing *Pd*. We found that the VOCs from these bacteria are complex and diverse (52). Notably, heptadecane was identified in 76.9% of the genera. Previous studies have shown that heptadecane produced by *Ba. amyloliquefaciens* can inhibit motility, biofilm formation, and root colonization by the tomato with pathogen *Ralstonia solanacearum* (53). Additionally, dimethyl disulfide was detected in 69.23% of the genera and is known for its broad antifungal activity (54). Sulfur-containing compounds, such as dimethyl sulfide, dimethyl disulfide, and dimethyl trisulfide, are effective against plant and human pathogens (55, 56) and strongly inhibit *Pd* pathogen (17). Our experiment also detected dimethyl sulfide, dimethyl disulfide, dimethyl trisulfide, 2-nonanone, nonanal, 2,5-dimethyl-pyrazine, 2-undecanone, 1-pentanol, 2-methyl-1-propanol, 1-undecene, and 2-methyl-1-butanol, which have been shown to inhibit *Pd* growth at certain concentrations (14, 15, 17, 19). While these VOCs have primarily been identified in bat skin microbiota, limited research has focused on VOCs produced by bacteria in bat roosting caves. Our study discovered that seven VOCs exhibited antifungal activity against *Pd* at a 100 mM concentration *in vitro*. Notably, 2-decanone released by avocado root bacteria completely inhibited the growth of *Fu. solani* hyphae, while 2-dodecanone inhibited approximately 40% of hyphal growth (57). Furthermore, 2-butyl-1-octanol detected in *St. levis* HFM-2 is believed to possess antibacterial, anticancer, and antioxidant activities (58). While our *in vitro* experiments identified potential VOCs to inhibit *Pd*, they should not be used directly on bats, as they may lose effectiveness once the fungus penetrates deeper tissues. Further research is needed to determine the minimum effective dose of VOCs and assess their ecological impacts on other cave species, including vertebrates, invertebrates, and microorganisms. Although our research focuses on caves in China, the findings could provide valuable insights for other regions, such as North America and Europe.

This study demonstrates that both the location within the cave and sampling time significantly affect the microbial community structure on cave walls. A major finding is that a considerable proportion of the cave microbiota exhibits anti-*Pd* activity. Importantly, the VOCs produced by these bacteria effectively inhibited *Pd* growth *in vitro*, making them promising candidates for probiotic development. By combining culture-independent and culture-dependent methods, we provide a more comprehensive characterization of microbial dynamics in bat hibernacula. However, a limitation of this study is the use of only two culture media, which may not fully capture the microbial diversity present. Future work employing media with varied nutrient compositions and pH levels could further elucidate the cave microbiome and its antifungal potential. Additionally, as this study focused on just one cave, expanding sampling to multiple hibernacula is essential to assess the generalizability of these findings. Future research should also investigate the inhibitory mechanisms of bacterial secondary metabolites (e.g., bacteriocins) against *Pd* in both *in vitro* and *in vivo* settings.

## MATERIALS AND METHODS

### Field sampling

On 13 December 2022, 17 February 2023, and 31 March 2023, we collected swab samples from the cave walls of the bat hibernaculum in New Cave in Jilin Province, China (Table S1). This single-entrance limestone cave, 223.97 m in length, was divided into distinct zones based on light exposure: the twilight zone and the dark zone. We measured cave wall temperatures during each sampling event using a Fluke 62 MAX IR Thermometer (Fluke, USA). For continuous microclimatic monitoring, three iButton temperature loggers (DS1922L, USA) were deployed year-round on dry cave walls, with two located in the dark zone and one in the twilight zone. Loggers were mounted on walls rather than the floor or ceiling to ensure stable temperature measurements. During the monitoring period, the cave’s temperature ranged from 7.2 to 16.4°C. Based on the cave’s structure and the locations where bats reside, 17 fixed sampling points were unevenly distributed between the twilight zone (*n* = 4) and the dark zone (*n* = 13) to cover as much of the bat habitat as possible, with the sampling points positioned independently of the bat’s habitat. The distance from the cave entrance ranged from 1.5 to 60 m, and the height above the ground ranged from 0.77 to 2.23 m. During sampling, three bat species were present: *Rhinolophus ferrumequinum*, *Murina leucogaster*, and *Myotis petax*, with population sizes ranging from 100 to 300 (Table 1).

**TABLE 1:** Number of samples collected^*a*^

Time	RNA later		30% glycerin	Bat species present
	Fungal DNA detection	Bacterial DNA detection	Culture and isolation	
13 Dec 2022	17 (13/4)	17 (13/4)	9 (6/3)	*R*. *ferrumequinum* (134); *My*. *petax* (2); *Mu*. *leucogaster* (60)
17 Feb 2023	17 (13/4)	17 (13/4)	9 (6/3)	*R*. *ferrumequinum* (159); *My*. *petax* (3); *Mu*. *leucogaster* (51)

^*a*^ The numbers in parentheses indicate the quantity of samples collected from the dark zone and the twilight zone in bat hibernacula; in the last column, values in parentheses indicate the number of bat individuals in bat hibernacula.

At each of the 17 sampling points, three dry sterile cotton swabs were used to swab the cave walls back and forth five times over a distance of 10 cm. Two swabs stored in RNA storage solution were used to extract fungal and bacterial genomic DNA, while the swab stored in 30% glycerol was used to isolate and culture cave wall microorganisms (Table 1). All samples were temporarily kept in an icebox and then preserved at −80°C for future analysis.

### DNA extraction, PCR amplification, and high-throughput sequencing of culture-independent communities

Genomic DNA was extracted from cave wall swabs using the DNeasy Blood & Tissue Kit (Qiagen, Hilden, Germany), with seven negative controls included to detect potential contamination (21). The samples were then analyzed using qPCR for *Pd* detection with primers nu-IGS-0169 (5′-TGCCTCTCCGCCATTAGTG-3′) and nu-IGS-0235 (5′-ACCACCGGCTCGCTACGTA-3′) on a StepOne instrument (Applied Biosystems, USA), following the protocol established by Muller et al. (59). Given the low *Pd* load in Chinese bats and caves, the qPCR was extended to 50 cycles (60). Each sample was tested in duplicate for accuracy, along with positive controls from *Pd* ATCC MYA-4855 (ATGC, USA). *Pd* load was calculated using the formula: log_10_(*Pd* (ng)) = (Ct − 22.04942)/ −3.34789, with the positive control set at 2 ng/μL (21). No contamination was detected in the negative controls. Samples with a Ct value greater than 40 or with no amplification were considered *Pd*-negative (61). The *Pd* isolates from China were confirmed to be *Pd* through sequencing, as detailed in the previous study by Hoyt et al. (5).

Total bacterial DNA was extracted from cave wall swabs using the FastDNA Spin Kit for Soil (MP Biomedicals, Santa Ana, CA, USA). The extracted DNA was then used as a template for PCR amplification of the V3–V4 region of the 16S rRNA gene using the primers 338F and 806R (62). The PCR products were purified, quantified, and sequenced on the Illumina Miseq PE300 platform at Majorbio Bio-Pharm Technology Co. Ltd. (Shanghai, China) for paired-end sequencing.

Quality control of the paired-end raw sequencing reads was performed using the software fastp (version 0.19.6, https://github.com/OpenGene/fastp) (63). Paired-end reads were merged using FLASH (version 1.2.11) (64). After quality control and merging, denoising of the sequences was carried out using the DADA2 plugin in QIIME2 (version 2022.08) (65), generating ASVs. Chimeric sequences, as well as chloroplast and mitochondrial sequences, were removed from all samples. A total of 3,253,145 reads were obtained from 51 samples (ranging from 52,111 to 120,119 reads per sample, with an average of 63,787). Denoising yielded a total of 30,033 ASVs across all samples.

To account for differences in sequencing depth across samples, alpha and beta diversity metrics were assessed using a repeated rarefaction approach following the method of Schloss (66). Alpha diversity metrics included Shannon diversity, Chao1 richness, and Faith’s phylogenetic diversity, while beta diversity metrics included Bray–Curtis dissimilarity and weighted and unweighted UniFrac distances. Sequencing depth was calculated for each sample, and all samples were subsampled without replacement to the minimum sequencing depth observed across samples (52,111 reads). Subsampling was performed using the rrarefy function in the vegan package, and the rarefaction procedure was repeated 1,000 times to account for stochastic variation introduced by subsampling.

For taxonomic composition analyses, ASVs with a mean relative abundance below 0.01% across all samples were excluded, leaving 11,847 ASVs for future analysis. Taxonomic classification was carried out using the Naïve Bayes classifier (67) in QIIME2, referencing the SILVAnr99 138 SSU database (68).

### Statistical analyses of culture-independent communities

Differences in *Pd* load, measured by quantitative PCR, across sampling times and areas within China were analyzed using Kruskal-Wallis tests on *Pd*-positive samples. Prior to performing the differential analysis, data were tested for normality and homogeneity of variance to meet the assumptions for parametric tests in groups with a sample size greater than two (*n* > 2).

Cave wall bacterial community composition at the phylum and genus levels was visualized for ASVs with a relative abundance greater than 0.01%. To identify microbial taxa associated with sampling time, sampling area, and *Pd* detection status, covariate-adjusted association analyses were performed using MaAsLin3 (Multivariate Association with Linear Models, v1.16.0) at the phylum and genus levels (69). Taxa with a global relative abundance below 0.01% across all samples were excluded prior to modeling. For each retained taxon, a linear model was fitted with relative abundance as the response variable and sampling time, sampling area, and *Pd* detection status as fixed effects. Relative abundances were normalized using total sum scaling (TSS) and log-transformed in MaAsLin3. *P* values were adjusted using the Benjamini–Hochberg false discovery rate (FDR), with associations considered statistically significant at *q* values ≤ 0.1.

To examine variation in alpha diversity across sampling time, area, and *Pd* detection status, we first assessed normality and homogeneity of variances using the Shapiro–Wilk and Bartlett tests, respectively (70, 71). If both tests indicated *P* values greater than 0.05, parametric tests (ANOVA for multiple groups and *t*-tests for pairwise comparisons) were applied. Otherwise, nonparametric tests (Wilcoxon or Kruskal-Wallis test) were used. To control the FDR in multiple comparisons, *P* values were adjusted using the BH method.

To examine differences in microbial community composition across sampling time, area, and *Pd* detection status, we performed Principal Coordinates Analysis (PCoA) for visualization. Homogeneity of dispersion among sample groups was assessed using PERMDISP via the betadisper() function, and Tukey’s test was applied to compare average dispersion between groups (72). Differences among groups were further tested using PERMANOVA with the adonis2() function in the vegan package. To account for potential pseudoreplication from repeated sampling, the analysis was stratified by sampling site ID (strata = SiteID), and the marginal effects of each factor were evaluated using the by = “margin” option with 9,999 permutations (73). *P* values were adjusted for multiple comparisons using the BH method.

To identify potentially pathogen-inhibiting taxa on cave walls, we focused on the top 30 bacterial genera by relative abundance for analysis and visualization, based on previously reported antifungal bacteria in the literature (14, 18, 38, 74–78) (Table S14). ASV sequences with a relative abundance > 0.01% were compared to published sequencing data using BLAST to evaluate sequence similarity. Variations in the relative abundance of these genera across sampling time, area, and *Pd* detection status were applied the same statistical approach used for the variation of alpha diversity analyses.

### Isolation and identification of culture-dependent bacterial communities

We conducted co-cultivation and isolation experiments using two types of media to maximize bacterial isolation. The Luria-Bertani (LB) agar, a nutrient-rich bacterial culture medium, contains 10 g NaCl, 5 g yeast extract, 10 g tryptone, 15 g agar, and 1,000 mL sterile water, with a pH of 7.2 ± 0.2. Reasoner’s 2A Agar (R_2_A), a low-nutrient medium suitable for slow-growing and stress-tolerant bacteria, contains 0.5 g yeast extract, 0.5 g tryptone, 0.5 g casein hydrolysate, 0.5 g glucose, 0.5 g soluble starch, 0.3 g K_2_HPO_4_, 0.024 g MgSO_4_, 0.3 g sodium pyruvate, 15 g agar, and 1,000 mL sterile water, with a pH of 7.2 ± 0.2. Samples preserved in 30% glycerol were streaked onto both media and incubated for three days. This incubation period was selected based on preliminary experiments and the growth characteristics of the target bacteria, which allowed sufficient time to observe most bacterial strains in this study. Bacterial colonies were isolated based on morphological characteristics, including shape, color, smoothness, transparency, and fluorescence. Pure strains were obtained after three rounds of sub-culturing. Following the protocol outlined by Li et al. (18), cultures were incubated under darkness at 13°C and 90% relative humidity to simulate cave microclimatic conditions.

For bacterial identification, the 16S rRNA gene was amplified using primers 27F and 1492R (18). The amplified DNA products were sent to Sangon Biotech Co., Ltd. (Shanghai, China) for Sanger paired-end sequencing. Sequences were merged and corrected using Geneious (version 2022.2.2). Taxonomic alignment was performed using both the NCBI tool (https://blast.ncbi.nlm.nih.gov/) and EZBioCloud (https://www.ezbiocloud.net/), according to the following criteria: (i) classify at the genus level if multiple matches exhibit a similarity greater than 99%; (ii) identify at the species level when only a single match had greater than 99%; (iii) identify at the genus level when multiple matches have a similarity of 100%; (iv) identify at the family level when all similarities are below 99%; and (v) if both databases provide a single 100% similarity match, identify at the genus level. In cases where the similarity differed between the two databases, the result with the highest similarity was selected for identification.

### Non-contact inhibition assays of culture-dependent bacterial communities

*Pd* spore suspensions were prepared following the method described by Lu et al. (17). Bacterial strains were cultured to the logarithmic stage to obtain bacterial solution. *Pd* spores were adjusted to a final concentration of 2 × 10^6^ spores, and all inhibition assays were incubated at 13°C and 90% relative humidity.

In the non-contact assay, 100 μL of the *Pd* spore suspension was spread onto each Sabouraud Dextrose Agar (SDA) medium, which contains 40.0 g of glucose, 10.0 g of peptone, 5.0 g of yeast extract, and 15.0–20.0 g of agar per liter of distilled water, with the pH adjusted to approximately 5.6. SDA is a widely used fungal growth medium that provides essential carbon and nitrogen sources for fungal growth (79). A 20 μL bacterial solution was inoculated onto LB solid medium. LB medium was used to ensure robust bacterial growth without altering the nutritional conditions of the fungal medium. Both Petri dishes were placed inside a larger 180 mm Petri dish, sealed with parafilm, and incubated for 14 days. This design excluded nutrient competition and direct interaction, thereby allowing specific assessment of inhibition mediated by bacterial-produced VOCs. The control group consisted of SDA plates inoculated with *Pd* spore only and incubated under identical conditions. Using ImageJ (version 1.8.0), the inhibition area was calculated according to the formula: inhibition rate (%) = (1 − area of experimental group / area of control group) × 100. The experimental group represents the area of *Pd* in the presence of bacteria, while the control group denotes the area of *Pd* in the absence of bacteria. The inhibition score of the non-contact experiment antagonistic bacteria on *Pd* is categorized as follows: 0 = negligible or minimal inhibition (below 50%) on *Pd*; 1 = significant inhibition on *Pd* (between 50% and 85%); or 2 = complete or nearly complete inhibition (above 85%) on *Pd* (14).

The bacterial solution was spread onto LB solid medium and cultured for 14 days, with uninoculated LB solid medium serving as a blank control. To identify VOCs produced by anti-*Pd* bacteria, gas chromatography-mass spectrometry (GC-MS) with headspace solid-phase microextraction was performed following the method described by Lu et al. (17). The VOC spectra were identified by comparison with the National Institute of Standards and Technology (NIST) database.

The VOCs detected in our study include some that have been identified in previous studies for their inhibitory effects against *Pd* (13–15, 17, 19, 80–82) (Table S13). In this study, we selected certain non-toxic VOCs with a relative abundance greater than 1% that were not previously tested for their inhibitory effects on *Pd* for further verification experiments. Based on the relative molecular mass and density of each VOC, stock solutions (100 mM) were prepared and serially diluted with 95% ethanol to obtain final concentrations of 10 mM and 1 mM. A 100 μL aliquot of *Pd* suspension was spread on SDA medium, and three sterile drug-sensitive tablets were placed onto the lid of the Petri dish. Then, 100 μL of VOC solutions at varying concentrations was applied to the drug-sensitive tablets and incubated for 14 days. The control group was treated with the corresponding volume of 95% ethanol. Each concentration of VOCs was tested in duplicate.

### Contact inhibition assays of culture-dependent bacterial communities

In the contact inhibition assay, *Pd* spores and bacteria were co-inoculated on the same SDA medium to evaluate inhibition via direct physical contact and diffusible non-volatile metabolites. A total of 100 μL of *Pd* spore suspension was spread onto SDA medium, followed by 20 μL of bacterial suspension onto the same plate in triplicate. The control group consisted of only 100 μL of *Pd* spore suspension on SDA medium. The inhibition rate was calculated using the same methodology as described above. The contact experiment inhibition score was calculated based on the inhibition area and categorized as follows: (i) strong inhibition for values ≥ 0.75, (ii) moderate inhibition for values < 0.75 but ≥ 0.50, (iii) weak inhibition for values < 0.50 but ≥ 0.25, and (iv) no inhibition for values < 0.25 (83). All the experiments were completed *in vitro*.

To clarify the proportion and relative abundance of ASVs in the culture-independent method compared to ASVs in the culture-dependent method, we utilized the align two or more sequences method using BLAST on NCBI. Specifically, we aligned the 16S rRNA data from the culture-dependent method with the sequencing data from the culture-independent method at the genus level, matching ASV sequences with 100% similarity.

## Data Availability

The raw sequence data were deposited to the Sequence Read Archive database of National Center for Biotechnology Information (NCBI) under BioProject accession numbers PRJNA1214429 and PRJNA1213301.

## SUPPLEMENTAL MATERIAL

Figure S1 aem.02214-25-s0001.tif Alpha diversity across different groups.

Figure S2 aem.02214-25-s0002.tif Barplot of group dispersions of cave wall microbial communities (PERMDISP).

Figure S3 aem.02214-25-s0003.tif Venn diagram of inhibitory strains identified by contact and non-contact assays.

Figure S4 aem.02214-25-s0004.tif *Pd*-inhibitory and non-inhibitory culture-dependent ASVs in cave wall communities revealed by 16S rRNA amplicon sequencing.

Figure S5 aem.02214-25-s0005.tif Upset diagram of VOCs detected by various genera.

Supplemental legends aem.02214-25-s0006.docx Legends for supplemental material.

Supplemental tables aem.02214-25-s0007.xlsx Tables S1 to S14.

ASM does not own the copyrights to Supplemental Material that may be linked to, or accessed through, an article. The authors have granted ASM a non-exclusive, world-wide license to publish the Supplemental Material files. Please contact the corresponding author directly for reuse.
