# Supplementary figures and images for: Role of anti-Pseudogymnoascus destructans bacteria in cave ecosystems during bat hibernation in northeast China
Source: Appl Environ Microbiol. 2026 Apr 24;92(5):e02214-25. doi: 10.1128/aem.02214-25 (PMC13188866; doi:10.1128/aem.02214-25)

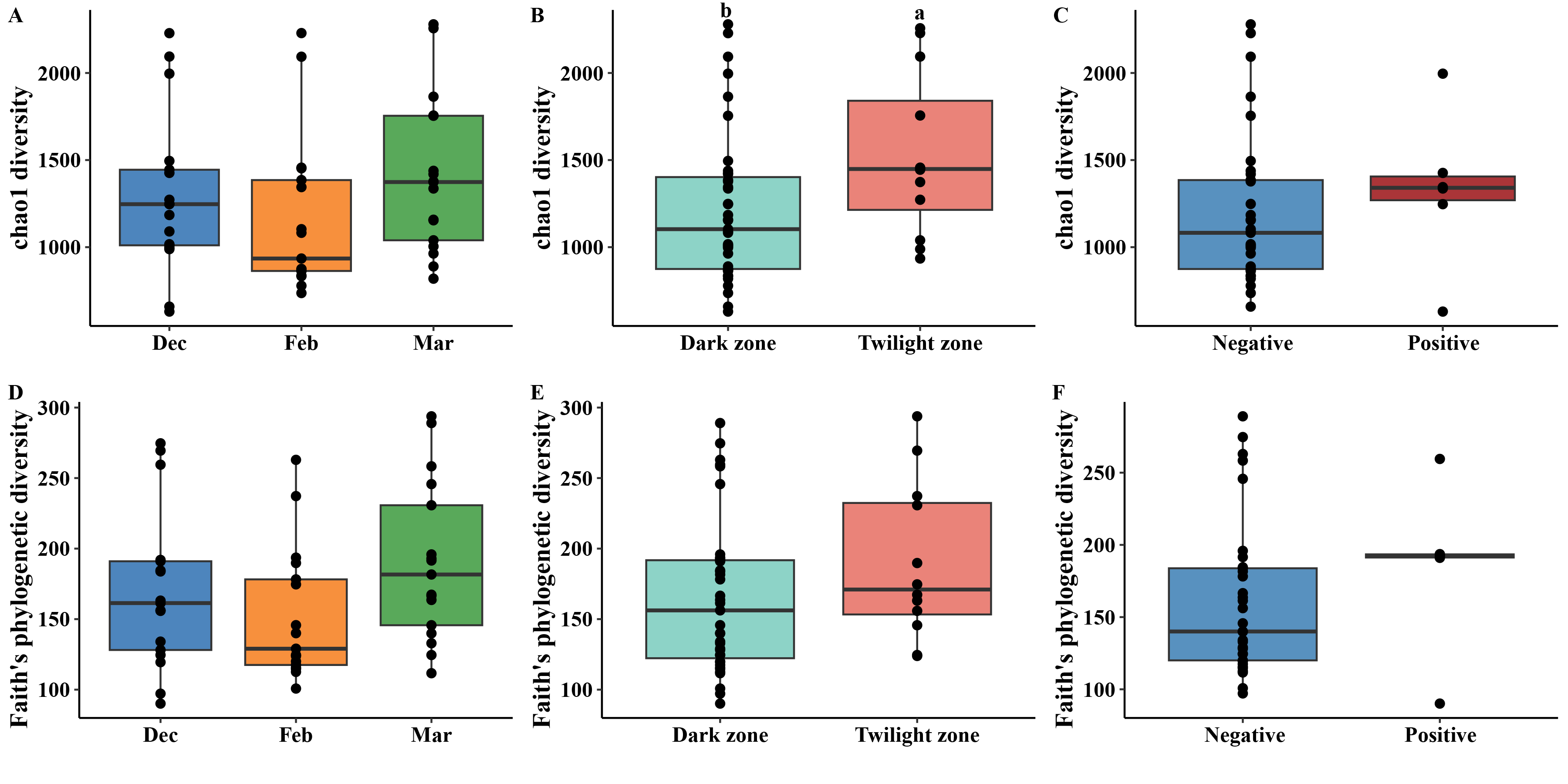

Supplement: Figure S1 — Alpha diversity across different groups. [file aem.02214-25-s0001.tif]

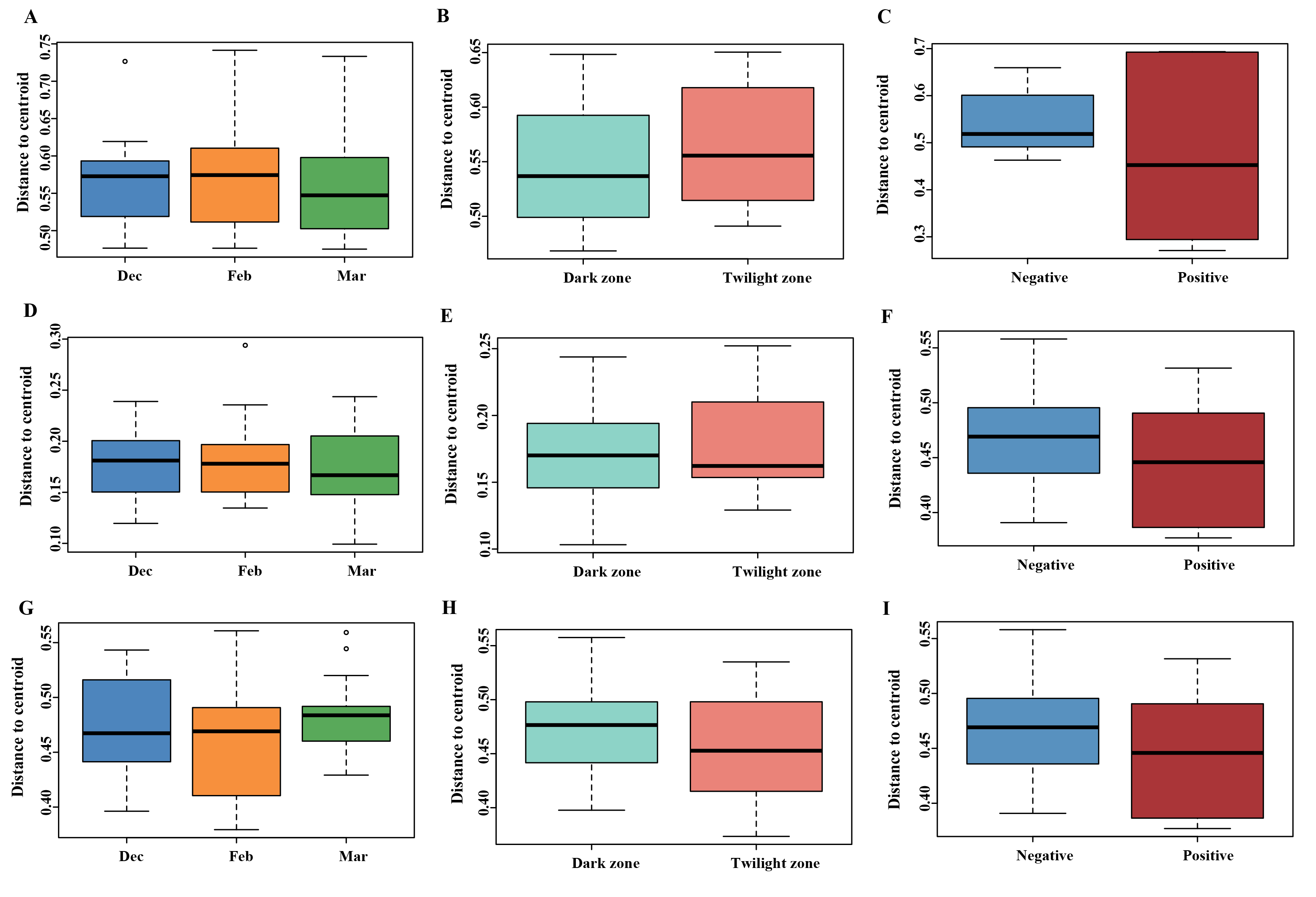

Supplement: Figure S2 — Barplot of group dispersions of cave wall microbial communities (PERMDISP). [file aem.02214-25-s0002.tif]

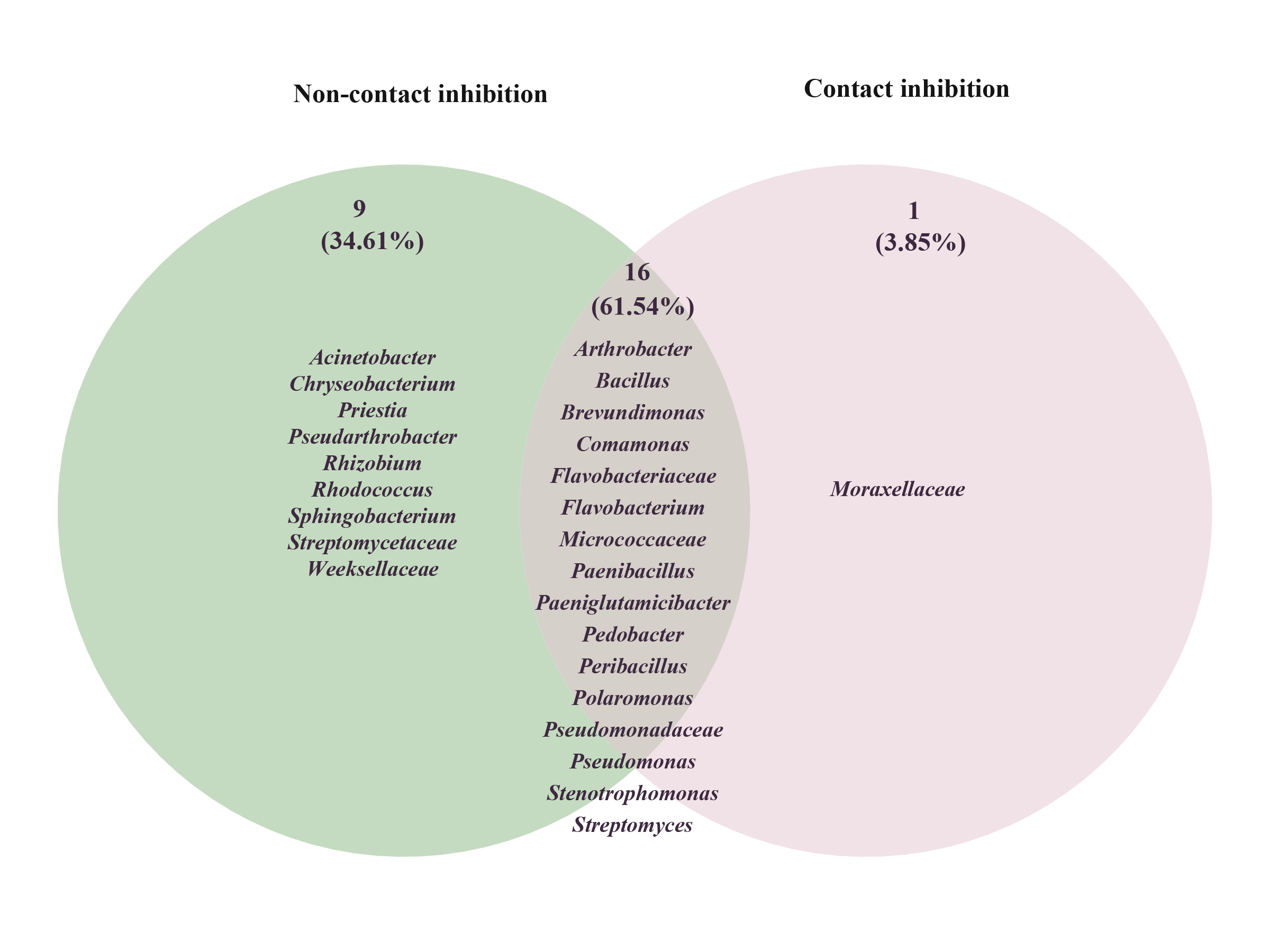

Supplement: Figure S3 — Venn diagram of inhibitory strains identified by contact and non-contact assays. [file aem.02214-25-s0003.tif]

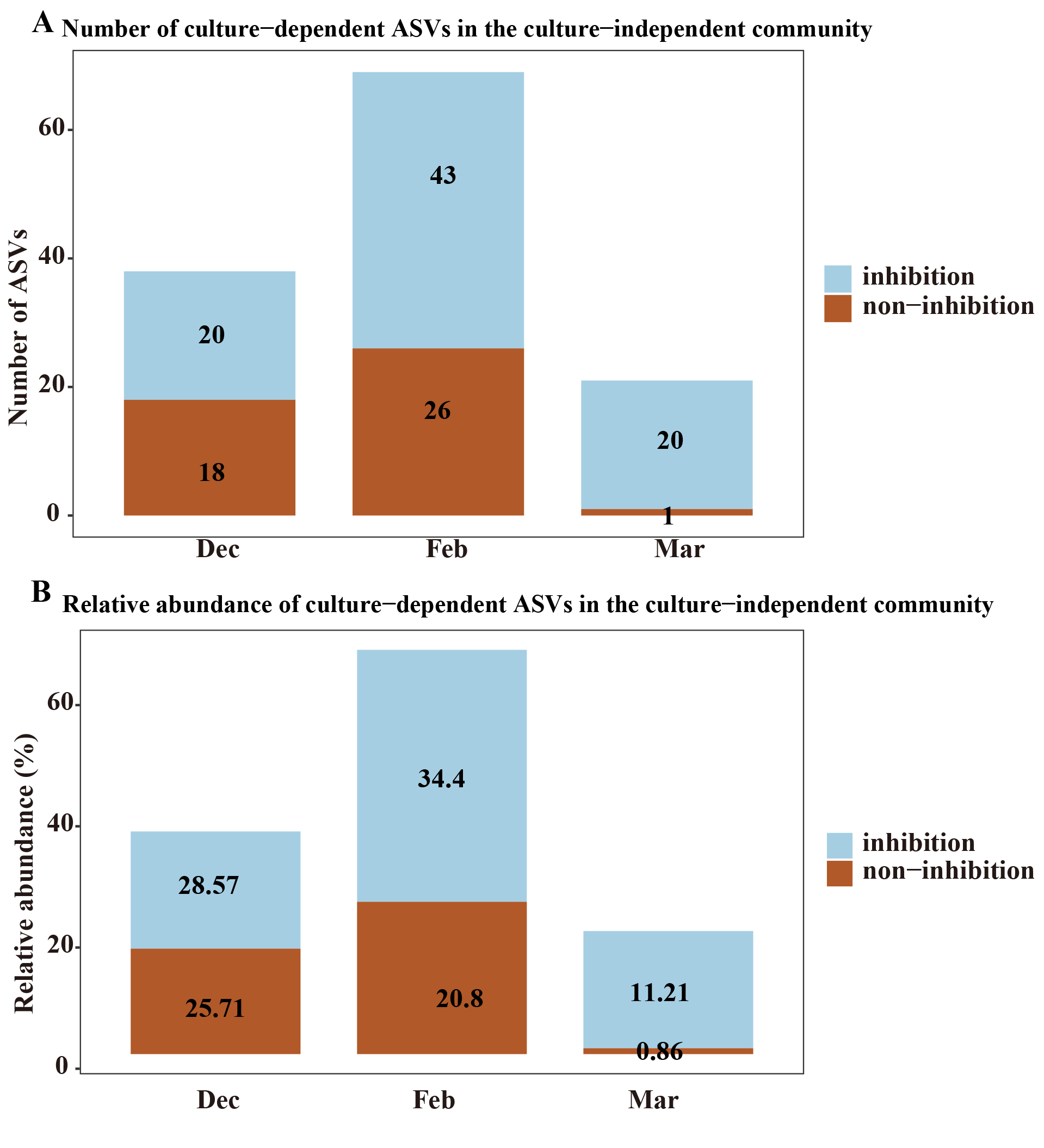

Supplement: Figure S4 — Pd-inhibitory and non-inhibitory culture-dependent ASVs in cave wall communities revealed by 16S rRNA amplicon sequencing. [file aem.02214-25-s0004.tif]

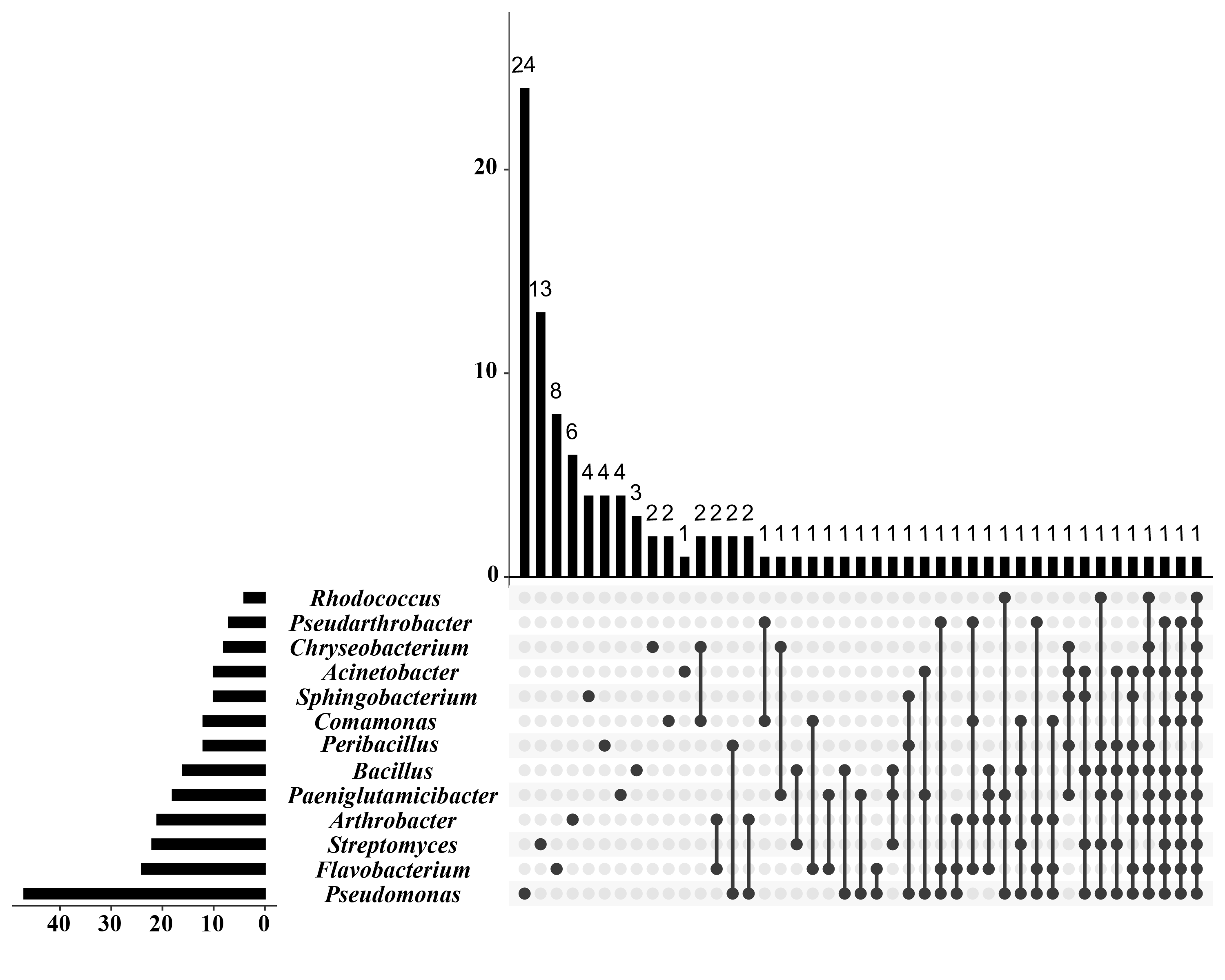

Supplement: Figure S5 — Upset diagram of VOCs detected by various genera. [file aem.02214-25-s0005.tif]
