# Supplementary material for: Role of anti-Pseudogymnoascus destructans bacteria in cave ecosystems during bat hibernation in northeast China
Source: Appl Environ Microbiol. 2026 Apr 24;92(5):e02214-25. doi: 10.1128/aem.02214-25 (PMC13188866; doi:10.1128/aem.02214-25)
Supplement: Supplemental legends — Legends for supplemental material. [file aem.02214-25-s0006.docx]

**Role of anti-*****Pseudogymnoascus destructans* bacteria in cave ecosystems during bat hibernation in Northeast China**

Leng Haixia ^a,b^, Sun Xiaoyu ^a,b^, Pu Yingting ^a,b^, Huang Long ^a,b^, Dai Wentao ^a,b^, Feng Jiang ^a,b,c,#^, Sun Keping ^a,b,c,#^

^a^Jilin Provincial Key Laboratory of Animal Resource Conservation and Utilization, Northeast Normal University, Changchun 130117, China.

^b^Key Laboratory of Vegetation Ecology, Ministry of Education, Changchun 130024, China.

^c^Jilin Provincial International Cooperation Key Laboratory for Biological Control of Agricultural Pests, Jilin Agricultural University, Changchun 130118, China.

* Address correspondence to Jiang Feng and Keping Sun, fengj@nenu.edu.cn; sunkp129@nenu.edu.cn

Running title: Anti-*Pd* Bacteria in Bat Hibernation Cave

The word count for the abstract: 205

**Supplementary materials**

**Supplementary Figure Legends**

Figure S1 Alpha diversity across different groups (sampling times, sampling areas, and *Pd* detection statuses). Letters indicate significant differences between groups, with the absence of letters indicating no significant differences.

**Figure S2 Box plots of multivariate homogeneity of group dispersions (variances) for cave wall microbial communities across different sampling times, sampling areas, and *Pd* detection statuses.** (A-C) show results based on Bray-Curtis distance, (D-F) on Weighted UniFrac distance, and (G-I) on Unweighted UniFrac distance.

**Figure S3 Venn diagram of strains with inhibitory effects in contact and non-contact inhibition.**

Note: Non-contact inhibition selects strains with inhibition scores of 1 and 2 against *Pd*, while contact inhibition selects strains with inhibition scores of 1,2, and 3 against *Pd*.

**Figure S4 Presence and relative abundance of *Pd* inhibition and non-inhibition, culture-dependent ASVs in the cave wall community obtained through 16S rRNA amplicon sequencing.** (A) Number of culture-dependent ASVs in the presence and absence of culture-independent communities. (B) Relative abundance of culture-dependent ASVs in the presence and absence of culture-independent communities.

**Figure S5 Upset diagram of VOCs detected by various genera.**

**Supplementary Table Legends**

Supplementary tables S1-S14 are provided in a separate Excel file.

**Table S1 Sampling, grouping, and *Pd* detection status in the cave walls.**

**Table S2 Environmental *Pd* load data were obtained from two publicly available datasets from North America.**

**Table S3 PERMANOVA analysis results of bacterial communities in different groups of cave walls.**

Note: R code used for PERMANOVA analysis is adonis2(~time+area+*Pd*_detection_status, data=group, permutations=9999, by="margin",strata = group$siteID)

**Table S4 Similarity of potential pathogen inhibition among ASVs with a relative abundance greater than 0.01% using culture-independent method.**

**Table S5 Comparing with the reference database, ASVs with a relative abundance greater than 0.01% that share 100% similarity.**

**Table S6 *P* values of significant differences in potential inhibition of *Pd* genera between different groups.**

**Table S7 Alignment results of culture-dependent sequences with two databases.**

**Table S8 Inhibitory effect of contact and non-contact inhibition experiments on *Pd* growth.**

Note: In non-contact inhibition experiments, 0 = negligible or minimal inhibition (below 50%) on *Pd*; 1 = significant inhibition on *Pd* (between 50% and 85%); or 2 = complete or nearly complete inhibition (above 85%) on *Pd*. In contact inhibition experiments, 1 = strong inhibition for values ≥ 0.75, 2 = moderate inhibition for values < 0.75 but ≥ 0.50, 3 = weak inhibition for values < 0.50 but ≥ 0.25, and 4 = no inhibition for values < 0.25.

**Table S9 Inhibition rate of non-contact and contact inhibition experiments.**

**Table S10 Comparison of culture-dependent isolates with culture-independent ASVs with a similarity of 100%.**

**Table S11 Composition of VOCs with a relative abundance greater than 1% detected by anti-*Pd* bacteria.**

**Table S12 VOCs used to validate the inhibitory effect on *Pd*.**

**Table S13 VOCs detected in this study with documented inhibitory activity against *Pd* in previous studies.**

**Table S14 Bacterial genera reported in previous studies to exhibit antifungal activity against fungal pathogens.**
